# Primary Amyloidosis with Gastric Involvement

**DOI:** 10.5005/jp-journals-10018-1113

**Published:** 2014-07-28

**Authors:** Serkan Borazan, Adil Coşkun, İrfan Yavaşoğlu, Yavuz Yeniçerioğlu, İbrahim Meteoğlu, Mehmet Hadi Yaşa, Ali Önder Karaoğlu

**Affiliations:** 1 Department of Internal Medicine, Division of Gastroenterology, Medical Faculty, Adnan Menderes University, Aydin, Turkey; 2 Division of Hematology, Medical Faculty, Adnan Menderes University, Aydin, Turkey; 3 Division of Nephrology, Medical Faculty, Adnan Menderes University, Aydin, Turkey; 4 Department of Pathology, Medical Faculty, Adnan Menderes University, Aydin, Turkey

**Keywords:** Amyloidosis, Gastrointestinal pathology, Implication of endoscopy.

## Abstract

Amyloidosis can involve all the segments of the gastrointestinal system (GIS) from mouth to anal canal. We present a case of amyloidosis that is detected by gastric biopsy taken in esophagogas-troduodenoscopy (EGD) performed to investigate the etiology of weight loss, nausea and vomiting. It is worth emphasizing that random gastric biopsy is important in gastric evaluations.

**How to cite this article:** Borazan S, Coşkun A, Yavaşoğlu İ, Yeniçerioğlu Y, Meteoğlu i, Yaşa MH, Karaoğlu AOÖ. Primary Amyloidosis with Gastric Involvement. Euroasian J Hepato-Gastroenterol 2014;4(2):107-109.

## INTRODUCTION

Amyloidosis can involve all the segments of the gastrointestinal system (GIS) from mouth to anal canal.^1^ Gastric involvement may appear as a component of primary amyloidosis (AL), secondary amyloidosis (AA), dialysis-dependent (p2-microglobulin) amyloidosis. Gastric involvement is seen rare in primary amyloidosis and can cause symptoms, such as upper gastrointestinal bleeding, abdominal pain, weight loss, nausea and vomiting.^2-4^ We present a case of amyloidosis that is detected by gastric biopsy taken in esophagogastroduodenoscopy (EGD) performed to investigate the etiology of weight loss, nausea and vomiting.

## CASE REPORT

**Figs 1A to D:**
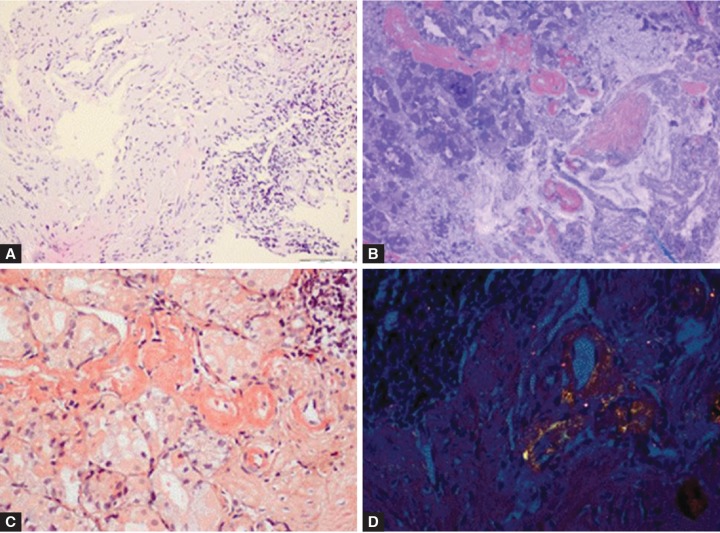
(A) Amorphous material accumulation in gastric biopsy specimen (H&E: 100x), (B) metachromatic staining with histochemical crystal violet stain (100x, crystal violet), (C) amyloid deposits on histochemical Congo red stain (200x, Congo red) and (D) yellow-green birefringence under a polarizing filter in amyloid deposit areas on histochemical Congo red stain (200x, Congo red)

A 67-year-old female patient was admitted with a history of weight loss of 16 kg in the last 1.5 months, and ongoing nausea and vomiting for the previous 10 days. She had a medical history of hypertension, coronary artery disease, congestive heart failure for 10 years and cholecystectomy in 2011. She was on olmesartan 20 mg/day, benidipine 4 mg/day, furosemide 40 mg/day, spironolactone 50 mg/day therapy and physical examination revealed no pathological findings except for a dry tongue and oral mucosa dryness. Laboratory findings were as hemoglobin 14.8 gm/dl, leukocyte count 9130/mm^3^, throm-bocyte count 348000/mm^3^, aspartate aminotransferase (AST): 24 U/L, alanine transaminase (ALT) 16 U/L, alkaline phosphatase (ALP) 54 U/L, gamma-glutamyl transferase (GGT) 41 U/L, total bilirubin 1 mg/dl, albumin 3.9 gm/dl, globulin 2.8 gm/dl, urea 108 mg/dl, creatinine 2.39 mg/dl, sodium: 136 mmol/l, potassium 4.9 mmol/l, C-reactive protein (CRP) 1.15 mg/l, erythrocyte sedimentation rate (ESR) 54 mm/h and 24-hour urine protein 3,094 mg, microalbumin 2,160 mg. The patient was hospitalized with diagnosis of acute renal failure. Abdominal ultrasonography was performed to investigate the etiology of nausea and vomiting, and shrunken kidneys were the only pathology detected; whereas reflux gastritis was diagnosed with EGD procedure. The amorphous material observed in the biopsy specimen taken from hyperemic, edematous corpus was treated with histochemical crystal violet and Congo staining, and amyloid accumulation was specified (Fig. 1).

**Fig. 2:**
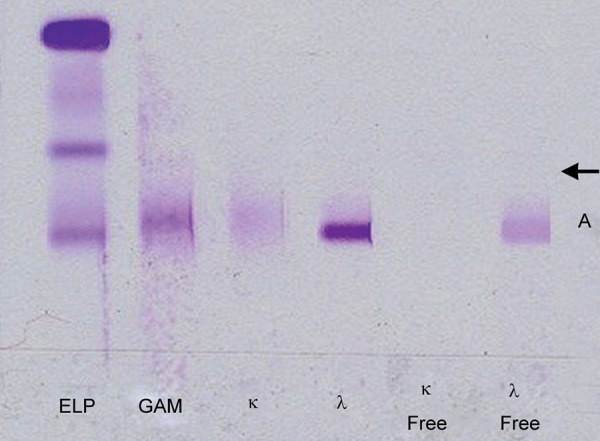
Urine immunoelectrophoresis

After diagnosis of amyloidosis, a rectoscopic examination was performed for subtype determination and the rectal biopsy material showed staining for lambda, and thus the findings were evaluated in favor of AL type (primary) amyloidosis. Bone marrow biopsy revealed 3% plasma cells; in histochemical examination, Congo red was positive. Serum immunoglobulins were found normal in immunoelectrophoresis; kappa 140 mg/dl, lambda 132 mg/dl, the kappa/lambda ratio 1:6. In urine immunoelectrophoresis, kappa 6.2 ng/dl and lambda 17.9 ng/dl (Fig. 2).

The patient was diagnosed with primary amyloi-dosis. Positron emission tomography/computed tomography (PET/CT) examination revealed mediastinal hypermetabolic lymph nodes and slightly hypermetabolic nodes in the left lung. In the peripheral blood, probrain natriuretic peptide (pro BNP) was 1832.1 pg/ml (normal: 0-100) and troponin I was 0.536 ng/ml (normal: 0-0.4) respectively. Echocardiogram showed the inter-ventricular septum width of 16 mm (normal: 0-11). The patient was diagnosed with primary amyloidosis with cardiac and gastric involvement and autologous bone marrow transplantation was planned.

The patient was diagnosed with primary amyloidosis through gastric, rectal, bone marrow biopsies and immuno-electrophoresis symptoms. Involvement of the gastrointestinal tract is very common in secondary amyloidosis (30-60%),^5^ while very rare in primary amyloidosis. In 769 patients diagnosed with primary amyloidosis, Menke et al reported gastrointestinal involvement in 8% of their patients and symptomatic gastric involvement only in 1°%.^2^ Epigastric pain, melena, weight loss, nausea and vomiting are the main symptoms in gastric involvement of primary amyloidosis.^6^ The mass may show up with a view of hemorrhage, erosion, ulcer and gastritis in the endo-scopic examination.^3,4^ As for our patient, whose plasma cell disease was not known previously, amyloidosis was detected in the gastric biopsy material taken during comprehensive investigation of the etiology of her weight loss, nausea and vomiting symptoms, which are the signs of gastric involvement. Through subsequent rectal biopsy and bone marrow samples, the patient was evaluated as primary amyloidosis. The kidney diminished in size was attributed to underlying hypertension. By further examinations, the patient was diagnosed with primary amyloidosis with cardiac and gastric involvement. In our case report, it was aimed to recall primary amyloidosis with gastric involvement, which is rarely identified in patients with symptoms, such as epigastric pain, melena, weight loss, nausea and vomiting (Table 1). It is worth emphasizing that random gastric biopsy is important in gastric evaluations in amyloidosis.

**Table 1:** **Table 1:** Chart of amyloidosis cases

*Reference*		*Age*		*Gender*		*Type*		*Site*	
Rivera et al^3^		*67*		Male		AL		Stomach	
Sawada et al^4^		72		Female		AL		Stomach	
Wu et al^6^		50		Female		AA		Stomach	
Our case		67		Female		AL		Stomach	
